# Anti‐SOX1 antibody‐positive paraneoplastic neurological syndrome presenting with Lambert‐Eaton myasthenic syndrome and small cell lung cancer: A case report

**DOI:** 10.1111/1759-7714.13290

**Published:** 2019-12-27

**Authors:** Chunyang Li, Xiaolei Wang, Lihua Sun, Hui Deng, Yanqiu Han, Wenqi Zheng

**Affiliations:** ^1^ Department of Neurology Affiliated Hospital of Inner Mongolia Medical University Hohhot China; ^2^ Department of Ultrasound Affiliated Hospital of Inner Mongolia Medical University Hohhot China; ^3^ Department of Clinical laboratory Affiliated Hospital of Inner Mongolia Medical University Hohhot China

**Keywords:** Anti‐SOX1antibody, Lambert‐Eaton myasthenic syndrome, paraneoplastic neurological syndromes, small cell lung cancer

## Abstract

Paraneoplastic neurological syndromes (PNS) are rare disorders affecting any part of the central, peripheral or autonomic nervous system that occur in association with cancer. Among cancer patients, less than 1% overall develop PNS. Anti‐SOX1 antibodies' positive paraneoplastic neurological disorders are rare and are usually associated with small cell lung cancer (SCLC). Here, we report a case of a 61‐year‐old male patient who presented with an unusual anti‐SOX1 positive PNS. The right tibialis anterior showed noticeable low‐amplitude motor unit potentials and high amplitude motor potentials in electrodiagnostic study, suggesting the presence of Lambert‐Eaton myasthenic syndrome (LEMS). Typical MRI and PET‐CT found a hyperintense lesion with contrast enhancement in the thorax in front of 5–6 centrum of vertebrae, and thoracoscopic biopsy revealed pathological findings for SCLC. The patient underwent several lines of chemotherapy and radiotherapy and survived for 15 months after the diagnosis of SCLC.

## Introduction

Paraneoplastic neurological syndrome (PNS) are rare immune‐mediated neurological disorders caused by the remote immune cross‐reaction between antigens, which are expressed by neurons and tumor cells.^1^ PNS can affect the underlying neoplasm as well as central, peripheral or autonomic nervous system and accounts for a constellation of clinical features.^2^ PNS of the central nervous system can appear as limbic encephalitis, paraneoplastic cerebellar degeneration (PCD), or opsoclonus‐myoclonus syndrome (OMS), whereas PNS of the peripheral nervous system can present as neuropathy, disorders of the neuromuscular transmission such as Lambert‐Eaton myasthenic syndrome (LEMS) or myasthenia gravis (MG).^3^, ^4^ Among cancer patients, less than 1% overall develop PNS. The most prominently associated tumor is small cell lung cancer (SCLC), and up to 3%–5% of PNS occurs with SCLC followed by other tumors such as lymphoid, breast, thymic and ovarian.^5^, ^6^, ^7^

In recent years, the detection of defined antineuronal autoantibodies has improved the diagnosis of PNS. Many of the well‐characterized antineuronal antibodies have been determined to be associated with PNS include the anti‐Hu, anti‐Yo antibody, or other increasing numbers of antineuronal autoantibodies such as Ma1, Ma2/Ta, CV2/CRMP5, and amphiphysin.^3^, ^8^ As an antiglial antibody associated with PNS, anti‐SOX1 antibody targets a Sry‐like high mobility group superfamily of developmental transcription factors preferentially expressed in the nuclei of Bergmann glia in the adult cerebellum.^9^, ^10^ It was initially found in the sera of SCLC patients without neurological disorders, but was absent in patients with other tumors in the reports by Gure *et al*. and Graus *et al*.^11^, ^12^ In addition in the report by Sabater *et al*., except for patients with a nonparaneoplastic LEMS, anti‐SOX1 antibody was detected in most patients with voltage‐gated calcium channel (VGCC) antibody‐associated LEMS indicating the presence of SCLC.^9^

Here, we report the case of a patient who presented to our clinic with neurological symptoms. Results of electro‐diagnostic studies, laboratory tests and pathological examination were consistent with a diagnosis of PNS with anti‐SOX1 antibody positive LEMS, and SCLC was subsequently diagnosed. The patient later developed generalized seizure and progressive confusion suggestive of limbic encephalitis (LE). This case shows the importance of a multidisciplinary team approach for the early recognition of PNS, and the importance of screening for additional autoantibodies in the presence of atypical symptoms. In addition, when neurological syndromes and paraneoplastic neuronal autoantibodies are present, an aggressive examine to rule out underlying cancer is important.

## Case report

The patient was a 61‐year‐old Chinese male, with a long history of drinking and a nonsmoker, who presented with a self‐reported three‐month bitter taste in the mouth causing some discomfort and unpleasantness during meals but no obvious dysphasia. In the following months, his gait gradually became unsteady and he developed weakness of the lower limbs. He had no variation in symptoms over the course of the day, but skeletal muscle fatigue increased. His neurological examination revealed proximal muscles of lower limb weakness at grade 4 on the Medical Research Council scale; poor deep tendon reflexes and heel‐knee‐shin test, and he had signs associated with cerebellar degeneration, such as dizziness, mild dysarthria, vertigo and clear ataxia. He displayed saccadic eye movements and nystagmus over the next few days, and developed the symptoms of diplopia. However, his cranial nerves and cognitive functions were normal. An evaluation was commenced that included laboratory study which was notable for positive antinuclear antibody (ANA) at 1:80 (reference <1:40), elevated serum ferritin (SF) at 509.6 ng/mL (reference 80–130 ng/mL), and positive treponema pallidum antibody (TP) at 9.85 (reference 0–1). However, venereal disease research laboratory test (VDRL) showed that reagin of cerebrospinal fluid (CSF) was negative. A CSF examination showed positive Pandy's test, and a raised level of total protein 0.824 g/L (reference 0.15–0.45), and an elevated IgG level of 72.1 mg/L (reference 6.3–33.3). The number of mononuclear cells was negative. In addition, CSF cytology revealed no atypical cells. Meanwhile, blood count, antineutrophil cytoplasmic antibodies (ANCA), biochemical analysis, erythrocyte sedimentation rate (ESR), renal and liver function, coagulation and serum electrolytes were within normal ranges.

**Figure 1:**
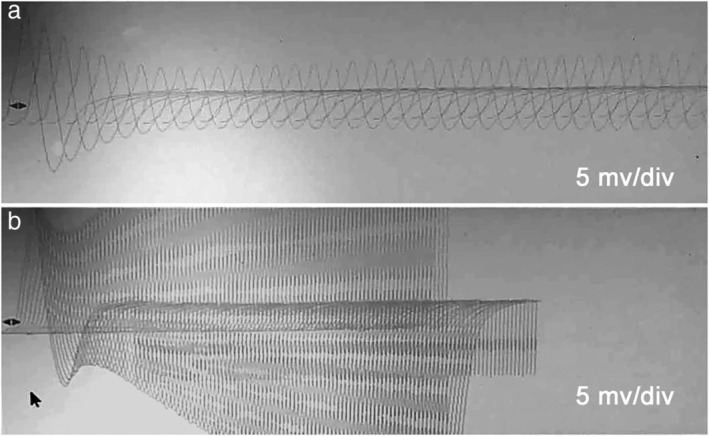
(**a**) Electromyography stimulation at 3 Hz showed a 55.9% decrement. Gain was 3 mV per division with a sweep speed of 5 ms. (**b**) There was an incremental response at high‐rate stimulation (50 Hz) for amplitude of 386% facilitation in area under the curve.

Myasthenia gravis serology testing showed that antistriated muscle antibodies and antimyocardium antibody were negative, and antiacetylcholine receptor (AchR) binding antibody was 0.01 nmol/L (reference negative < 40 nmol/L; equivocal 40 nmol/L–50 nmol/L; >positive 50 nmol/L); however, the anti‐SOX1 antibody was positive, confirming the presence of LEMS. The electrodiagnostic study showed notable low amplitude motor potentials of the right tibialis anterior and left trapezius, and repetitive nerve stimulation (RNS) at low frequency (3 Hz) elicited a decremental response. Meanwhile, the right tibialis anterior was tested with high‐frequency (50 Hz) RNS, where 386% facilitation was observed, consistent with LEMS (Fig 1).

**Figure 2:**
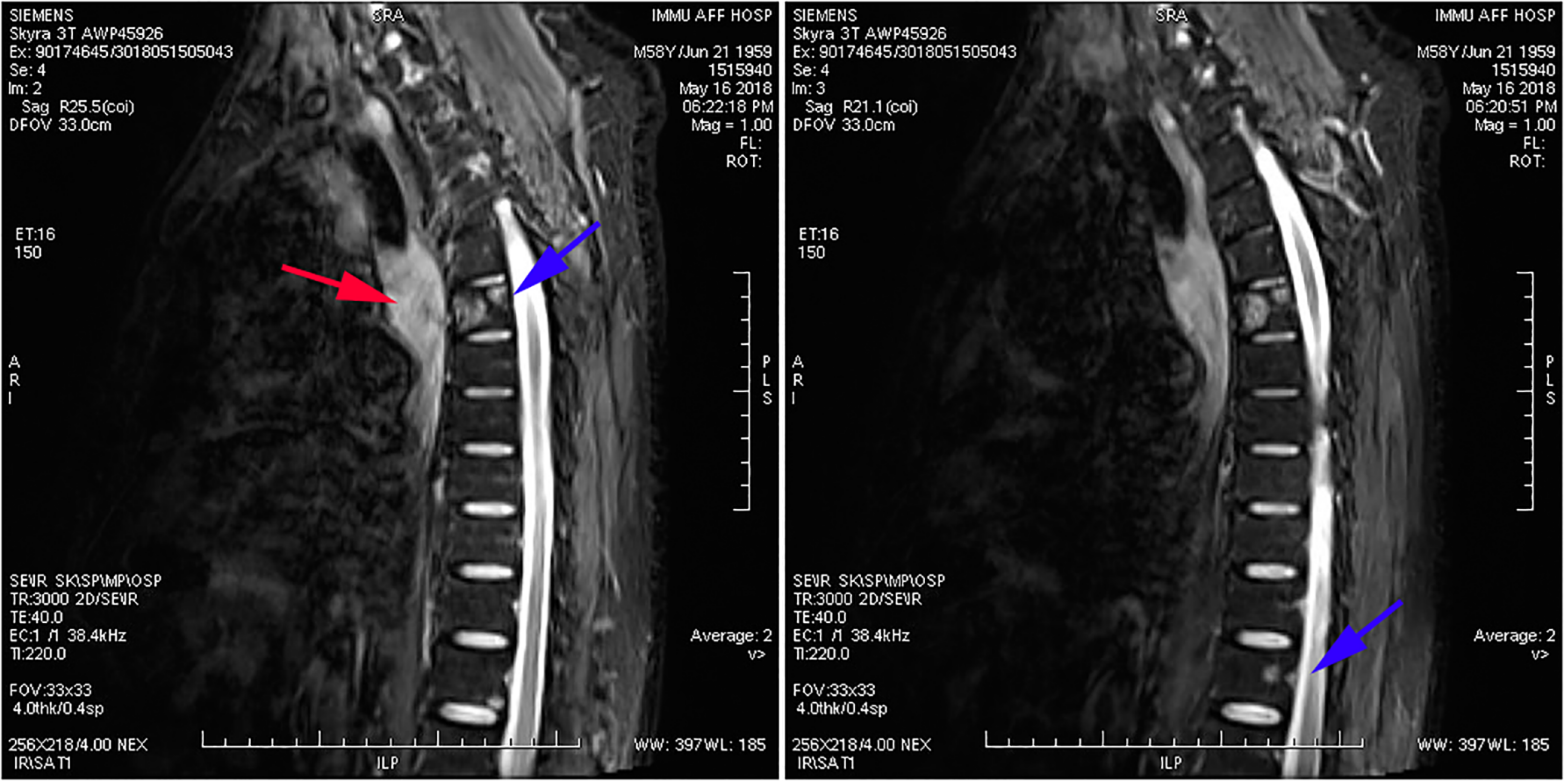
Spinal MRI demonstrated remarkable enhancement of the thorax in front of 5–6 centrum (as indicated by the red arrow), and revealed T6, and T12 destructive vertebral body lesions (indicated by blue arrows).

**Figure 3:**
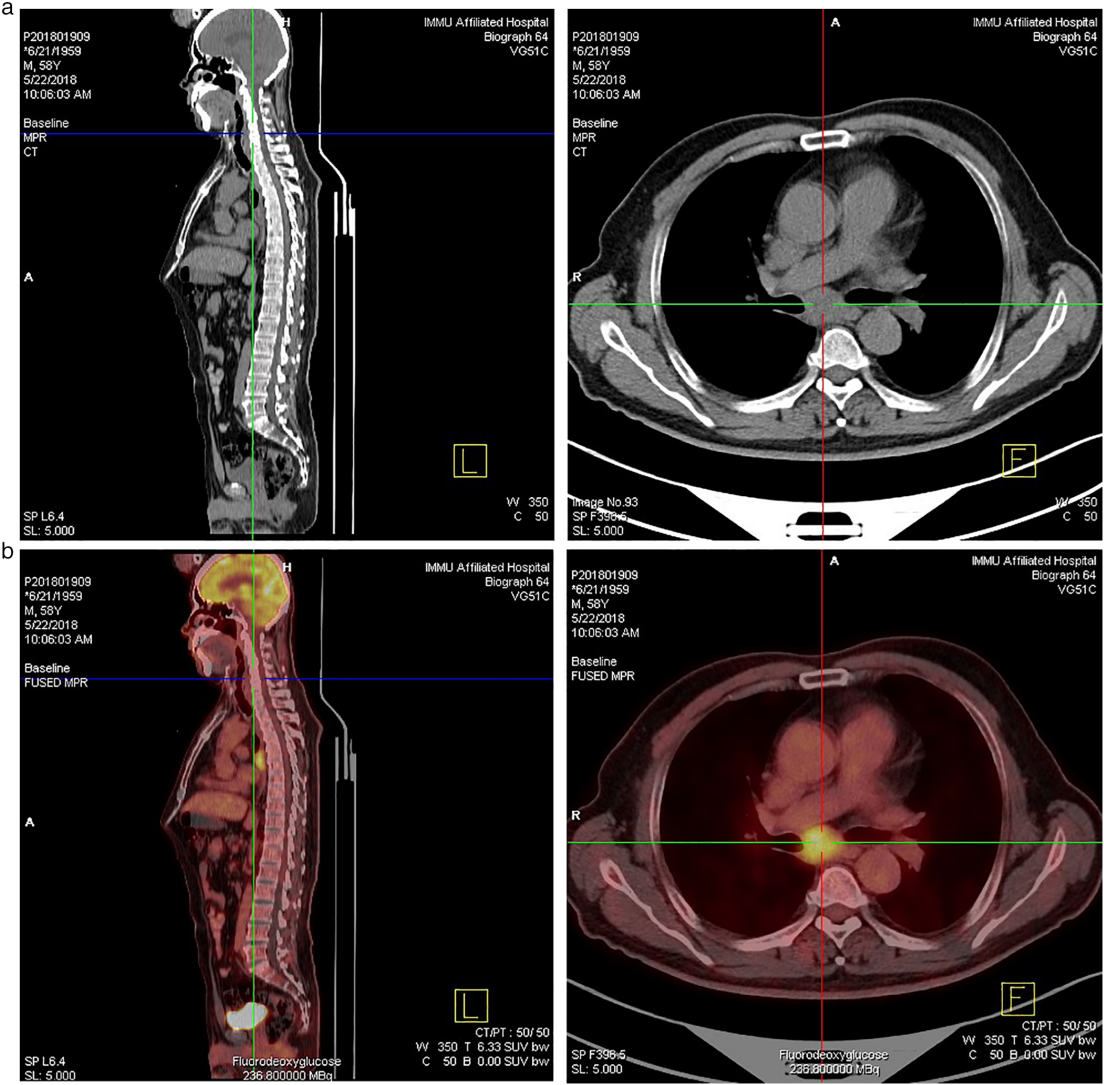
(**a**) PET‐CT showed multiple enlarged lymph node shadows associated with increased metabolism in the anterior tracheal vena cava, right pulmonary hilum and subcarina. (**b**) [(18)F] fluoro‐2‐deoxy‐D‐glucose positron emission tomography/computed tomography imaging revealed an area of abnormal FDG accumulation in the mediastinum.

The patient began investigational treatment with pyridostigmine bromide tablets for one week (60 mg, every 8 hours, per os), and the symptoms of diplopia disappeared although he still felt weak. Soon afterwards, he developed several days of progressive confusion and psychiatric symptoms suggestive of LE. Well characterized onconeural antibodies including Hu‐Ab, Ri‐Ab, Tr‐Ab, GAD‐Ab, Yo‐Ab, CV2‐Ab, ANNA‐3‐Ab, PCA2‐Ab, Ma2‐Ab, and anti Amphiphysin‐Ab all tested negative. Since magnetic resonance imaging (MRI) (Fig 2) and PET‐CT (Fig 3a,b) demonstrated hyperintense lesions with contrast enhancement of the thorax in front of 5–6 centrum, anti‐SOX1 antibody‐positive PNS associated with SCLC was suspected. Brain MRI shown no brain metastases, and no cerebellar or limbic alterations. A subsequent thoracoscopic biopsy of the lung tissue was performed in May 2018 at the Chinese PLA General Hospital which revealed pathological findings of SCLC in the mediastinal lymph nodes. A total of four cycles of chemotherapy (60 mg/m^2^ cisplatin on day 1, and 60 mg/m^2^ irinotecan on days one, eight, and 15) and radiotherapy (total 36 Gy) were administered, followed by thoracoscopic lobectomy three weeks after his last chemotherapy at the Chinese PLA General Hospital. His neurological symptoms did not significantly improve, and his hospital course was complicated by acute hypercapnic respiratory and supraventricular tachycardia in August 2019, culminating in a cardiac arrest. As the patient was “do not resuscitate/do not intubate” (DNR/DNI), he died 15 months after the diagnosis of SCLC.

## Discussion

Here, we report a case of anti‐SOX1 associated PNS presenting with LEMS and LE in a patient with SCLC tumor. Our case highlights several characteristic features of anti‐SOX1 associated PNS, including “classical” paraneoplastic neurological syndromes, with well‐characterized onconeural antibodies and pathologically determined cancer. PNS occurs with increased frequency in patients with cancer. They are often therapy‐refractive and relentlessly progressive. In the clinical features of “classical” PNS, the most relevant advance is LEMS. LEMS occurs as a paraneoplastic neurological syndrome in approximately 50%–60% of the cases.^3^, ^13^ As a rare autoimmune disorder of neuromuscular junction transmission, LEMS often presents with clinical features of proximal muscle weakness, diminished deep tendon reflexes, and autonomic dysfunction, and the associated tumor is almost solely SCLC.^14^ As an aggressive tumour of neuroectodermal origin, SCLC is most strongly associated with a history of smoking. Although SCLC is initially responsive to chemotherapy, the tumor is often fatal with a median survival from diagnosis of only eight to 10 months. More rarely, as in our patient with SCLC who was a nonsmoker, there were no symptoms of weight loss. Although the patient had a long history of heavy drinking, he had been sober for almost a year when he first came to our hospital. The SCLC induction reason of this patient remains unclear.

In most cases, the manifestations of PNS occur before the diagnosis of pulmonary carcinoma, and always antedate its diagnosis.^8^ Furthermore, given the fact that occurrences of PNSs are rare, many cases are not readily diagnosed as PNS. Identifying antineuronal antibodies is crucial. Even if several associated antibodies have been reported, fewer than 50% of patients with PNS harbor paraneoplastic antibodies.^3^ Anti‐SOX1 antibodies, also known as antiglial nuclear antibody (AGNA), are specifically found in paraneoplastic neurological disorders. Since SOX1 is expressed in neuronal precursor cells in the developing central nervous system, it has been used as an early marker of neural stem cells.^15^ Anti‐SOX1 antibodies are most often associated with SCLC.^16^ In a recent study, positive SOX1 antibodies were detected in 64% of patients with LEMS and SCLC, in 32% of patients with PNS and SCLC, and in 22% of patients with SCLC alone.^9^, ^17^ In our case, routine antibodies to PNS such as Hu‐Ab, Ri‐Ab, Tr‐Ab, GAD‐Ab, Yo‐Ab were negative, but SOX1 was strongly positive, and this finding encourages the testing of comprehensive onconeural antibodies. Also, when neurologists evaluate a patient with LEMS, routine screening for an underlying tumor should be carried out.

## Disclosure

The authors declare that they have no competing interests.

## References

[1] Voltz R . Paraneoplastic neurological syndromes: An update on diagnosis, pathogenesis, and therapy. Lancet Neurol 2002; 1 (5): 294–305. 12849427 10.1016/s1474-4422(02)00135-7

[2] Viaccoz A , Honnorat J . Paraneoplastic neurological syndromes: General treatment overview. Curr Treat Options Neurol 2013; 15 (2): 150–68. 23436113 10.1007/s11940-013-0220-2

[3] Graus F , Delattre JY , Antoine JC e a . Recommended diagnostic criteria for paraneoplastic neurological syndromes. J Neurol Neurosurg Psychiatry 2004; 75 (8): 1135–40. 15258215 10.1136/jnnp.2003.034447 PMC1739186

[4] Dalmau JO , Posner JB . Paraneoplastic syndromes affecting the nervous system. Semin Oncol 1997; 24 (3): 318–28. 9208887

[5] Kanaji N , Watanabe N , Kita N e a . Paraneoplastic syndromes associated with lung cancer. World J Clin Oncol 2014; 5 (3): 197–223. 25114839 10.5306/wjco.v5.i3.197 PMC4127595

[6] Lorusso L , Hart IK , Ferrari D , Ngonga GK , Gasparetto C , Ricevuti G . Autonomic paraneoplastic neurological syndromes. Autoimmun Rev 2007; 6 (3): 162–8. 17289552 10.1016/j.autrev.2006.10.003

[7] Rees JH , Hain SF , Johnson MR *et al* The role of [18F]fluoro‐2‐deoxyglucose‐PET scanning in the diagnosis of paraneoplastic neurological disorders. Brain 2001; 124 (Pt 11): 2223–31. 11673324 10.1093/brain/124.11.2223

[8] Darnell RB , Posner JB . Paraneoplastic syndromes involving the nervous system. N Engl J Med 2003; 349 (16): 1543–54. 14561798 10.1056/NEJMra023009

[9] Sabater L , Titulaer M , Saiz A , Verschuuren J , Gure AO , Graus F . SOX1 antibodies are markers of paraneoplastic Lambert‐Eaton myasthenic syndrome. Neurology 2008; 70 (12): 924–8. 18032743 10.1212/01.wnl.0000281663.81079.24

[10] Sottile V , Li M , Scotting PJ . Stem cell marker expression in the Bergmann glia population of the adult mouse brain. Brain Res 2006; 1099 (1): 8–17. 16797497 10.1016/j.brainres.2006.04.127

[11] Gure AO , Stockert E , Scanlan MJ e a . Serological identification of embryonic neural proteins as highly immunogenic tumor antigens in small cell lung cancer. Proc Natl Acad Sci U S A 2000; 97 (8): 4198–203. 10760287 10.1073/pnas.97.8.4198 PMC18195

[12] Graus F , Vincent A , Pozo‐Rosich P e a . Anti‐glial nuclear antibody: Marker of lung cancer‐related paraneoplastic neurological syndromes. J Neuroimmunol 2005; 165 (1‐2): 166–71. 15949849 10.1016/j.jneuroim.2005.03.020 PMC2586939

[13] Titulaer MJ , Lang B , Verschuuren JJ . Lambert‐Eaton myasthenic syndrome: From clinical characteristics to therapeutic strategies. Lancet Neurol 2011; 10 (12): 1098–107. 22094130 10.1016/S1474-4422(11)70245-9

[14] Elrington GM , Murray NM , Spiro SG , Newsom‐Davis J . Neurological paraneoplastic syndromes in patients with small cell lung cancer. A prospective survey of 150 patients. J Neurol Neurosurg Psychiatry 1991; 54 (9): 764–7. 1659614 10.1136/jnnp.54.9.764 PMC1014512

[15] Wegner M . From head to toes: The multiple facets of Sox proteins. Nucleic Acids Res 1999; 27 (6): 1409–20. 10037800 10.1093/nar/27.6.1409 PMC148332

[16] Graus F , Saiz A , Dalmau J . Antibodies and neuronal autoimmune disorders of the CNS. J Neurol 2010; 257 (4): 509–17. 20035430 10.1007/s00415-009-5431-9

[17] Vural B , Chen LC , Saip P e a . Frequency of SOX Group B (SOX1, 2, 3) and ZIC2 antibodies in Turkish patients with small cell lung carcinoma and their correlation with clinical parameters. Cancer 2005; 103 (12): 2575–83. 15880380 10.1002/cncr.21088

